# Re: Letter to the Editor in Response to ‘Molecular detection of rabies virus strain with N-gene that clustered with China lineage 2 co-circulating with Africa lineages in Monrovia, Liberia: first reported case in Africa’

**DOI:** 10.1017/S0950268819001973

**Published:** 2019-12-11

**Authors:** Ayodeji Oluwadare Olarinmoye, Varney Kamara, Nykoi Dormon Jomah, Babasola Oluseyi Olugasa, Olayinka Olabisi Ishola, Abrahim Kamara, Pam Dachung Luka

**Affiliations:** 1 Engineer Abdullah Bugshan Research Chair for Dental and Oral Rehabilitation (DOR), King Saud University, Riyadh, Saudi Arabia; 2 Center for Control and Prevention of Zoonoses (C.C.P.Z.), Faculty of Veterinary Medicine, University of Ibadan, Ibadan, Nigeria; 3 Ministry of Agriculture, Leon Quest Ledlum Central Veterinary Diagnostic Laboratory, Fendel, Monrovia, Liberia; 4 Central Agricultural Research Institute (C.A.R.I.), Suakoko, Bong County, Liberia; 5 Department of Veterinary Public Health and Preventive Medicine, University of Ibadan, Ibadan, Nigeria; 6 Ministry of Agriculture Sub-office, Buchanan, Grand Bassa County, Liberia; 7 National Veterinary Research Institute (N.V.R.I.), Vom, Plateau State, Nigeria

Sir,

We are pleased that our article has stimulated a letter to the Editor by Zhao and colleagues, and that they have contributed to the goal of stepwise action for improving rabies surveillance in Liberia. The goal of our study was to improve rabies surveillance in Liberia, towards stepwise elimination of the disease in the country by 2030. At the onset of the study, the exact species and strains of lyssaviruses responsible for animal and human rabies in Liberia were unknown. However, because a majority of the documented clinical rabies cases in the human population of the country had underlying dog bite histories [1–3], we hypothesised that dog-adapted Rabies lyssavirus (RABV) strains are responsible for enzootic rabies in Liberia [4]. We confirmed this with our report of the **first GenBank deposition** of a RABV from Liberia, and it was a transcontinental strain of RABV genotype 1 (GenBank accession number: MF765758), co-circulating with Africa lineages in the national capital, Monrovia [4]. Phylogenetic analysis of the partial N gene of MF765758 (554 bp) revealed its very close resemblance with the China lineage 2 RABV strains KU963489 (or SN2-62-CanineCHINA2005) and DQ666322 (or Jiangsu_Yc63) previously isolated from dogs in China, and other RABV lineages including Africa 1A–1C, Africa 4, Europe/Middle East and Asia, and the vaccine strains PV (GU992322), SRV9 (AF499686) and SAD Vnukovo (GU992319) [4].

**Fig. 1.**
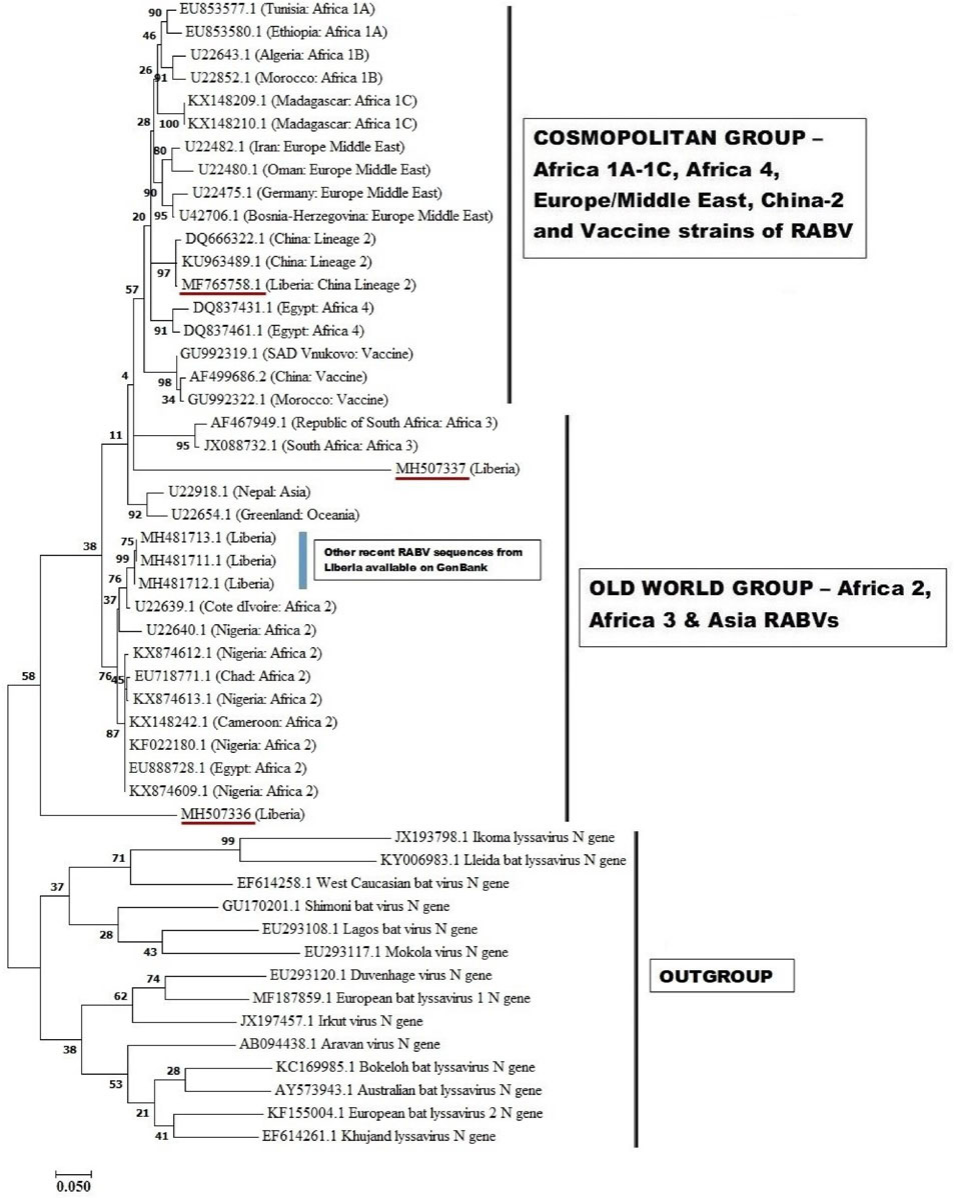
Maximum likelihood phylogenetic tree of the nucleoprotein (N) gene of MF765758. The analysis was performed using MEGA7 and involved 50 nucleotide sequences. The tree is drawn to scale, with branch lengths in the same units as those of the evolutionary distances used to infer the phylogenetic tree. The evolutionary distances were computed using the Kimura 2 parameter model and are in the units if base substitutions per site.

In their letter, Zhao and colleagues similarly observed that apart from the two RABV strains of China lineage 2 (KU963489 and DQ666322), MF765758 also has very close resemblance (99% similarity) to other RABVs such as the France RABV strain, CVS (GU992321), the India RABV strains, RAB5 and RAB7 (KF535200 and KF535201), and that phylogenetic analysis ‘revealed them to be the same lineage’. This statement corroborates our ealier conclusion that phylogenetic evidence suggests that, ‘the Monrovia RABV isolate (MF765758) has its origin in Asia, rather than it being an autochthonous (Liberian) RABV or an extant strain from another African country’ [4]. In addition, we designated as **cosmopolitan**, the group of RABVs from across the world that have the same progenitor as MF765758 (Fig. 1) [4]. Previously, Vellasco Villa and others had similarly described a cosmopolitan group of RABV lineages comprising dog-maintained and dog-derived strains belonging to Africa 1 and 4, North America, South America, Europe, Middle East and vaccine lineages [5].

**Fig. 2.**
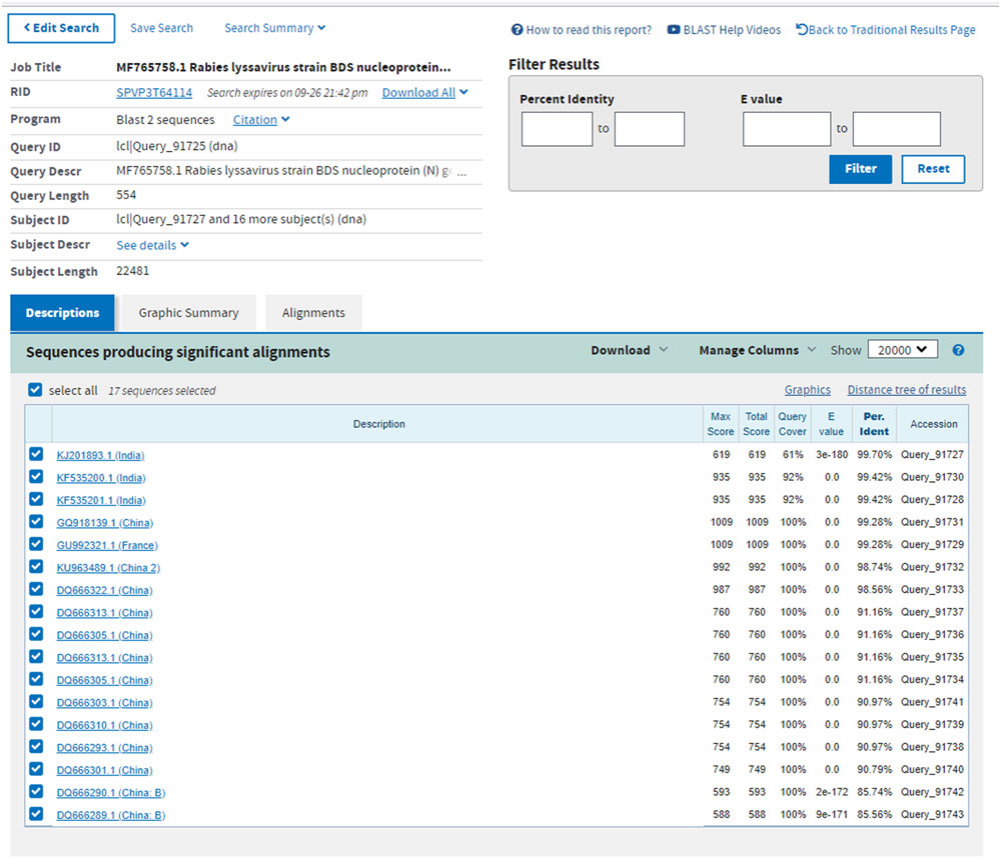
Multiple sequence alignment of the N gene of MF765758 and those of selected Rabies lyssaviruses obtained from the GenBank.

We appreciate the inclusion of the France RABV strain CVS (GU992321) and the India RABV strains RAB5 and RAB7 (KF535200 and KF535201) and others in the BLAST analysis of MF765758, by Zhao and colleagues. This has added to the knowledge base of the phylogeny of MF765758 and further unravelled the mystery of how MF765758 arrived in Liberia. However, because of the lower *E* values and percentage query cover assigned to Indian RABVs KJ201893, KF535201 and KF535200 when aligned with MF765758, as compared with China lineage C RABVs (Fig. 2), there is a need to exercise caution in the interpretation of the BLAST results that show that some of these RABVs (particularly those that were isolated in India), have a higher resemblance to MF765758 than do the Chinese C2 RABVs DQ666222 and KU963489. We agree with Zhao and colleagues, on the need for sequence analysis of the full length of the nucleoprotein (N) and glycoprotein (G) genes of MF765758, as this would further help to resolve this issue.

**Fig. 3.**
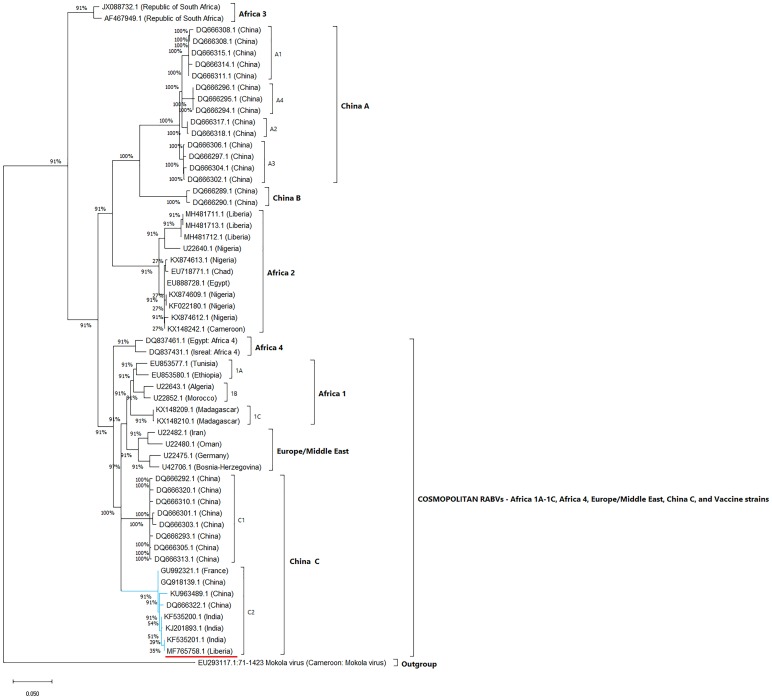
Maximum likelihood phylogenetic tree of the nucleoprotein (N) gene of MF765758. The analysis was performed using MEGA X and involved 58 nucleotide sequences. The tree is drawn to scale, with branch lengths in the same units as those of the evolutionary distances used to infer the phylogenetic tree. The evolutionary distances were computed using the Kimura 2 parameter model and are in the units of base substitutions per site.

**Fig. 4.**
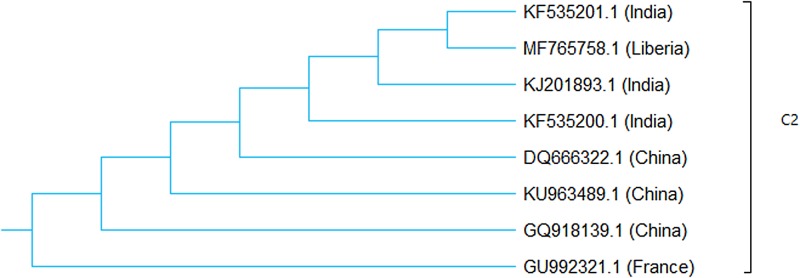
Maximum likelihood phylogenetic sub-tree of MF765758.

Three genetically clustered groups of RABVs (A–C) were recently described, based on the characterisation of RABV nucleoprotein genes in dogs in Southeast China [6]. Phylogenetic analysis of MF765758 revealed its segregation along with the Indian RABVs KJ201893, KF535201 and KF535200, and China C2 RABVs DQ666222 and KU963489 (Figs 3 and 4). We consider this statement to be a confirmation of our earlier conclusion that these RABV strains are offshoots of the same node of the phylogenetic tree. We posited that ‘The co-circulation of China lineage-2 with Africa lineage-2 and Africa lineage-3 RABVs in Monrovia, Liberia was probably due to inadvertent importation of rabies virus into Liberia from China or neighbouring countries, as a result of detection failure along import barriers [4]’. The predominance of China sourced rabies vaccines in Nigeria [7], the all-time high Chinese involvement in post-conflict reconstruction efforts in Liberia [4], and rabies vaccine quality compromise in China with resultant rabies outbreak following dog bite in humans [8, 9] were additional factors that we considered in explaining the likely origin of MF765758. We deem to be of vital importance to the unravelling of the historical biogeography of MF765758, the molecular clock analysis or divergence time estimation of the individual members of the China C2 group of RABVs.
